# Effects of task-oriented robot training on arm function, activity, and quality of life in chronic stroke patients: a randomized controlled trial

**DOI:** 10.1186/1743-0003-11-45

**Published:** 2014-03-31

**Authors:** Annick AA Timmermans, Ryanne JM Lemmens, Maurice Monfrance, Richard PJ Geers, Wilbert Bakx, Rob JEM Smeets, Henk AM Seelen

**Affiliations:** 1 Adelante, Centre of Expertise in Rehabilitation and Audiology, Hoensbroek, The Netherlands; 2 Research School CAPHRI, Department of Rehabilitation Medicine, Maastricht University, Maastricht, The Netherlands; 3 Adelante Rehabilitation Centre, Hoensbroek, The Netherlands; 4 Department of Rehabilitation Medicine, Maastricht University Medical Centre, Maastricht, The Netherlands; 5 Reval - Rehabilitation Research Institute, Biomed – Biomedical Research Institute, Faculty of Medicine and Life Sciences, Hasselt University, Belgium

**Keywords:** Stroke, Hemiplegia, Robotics, Computer-assisted therapy, Rehabilitation, Upper extremity, Arm, Hand, Motor skills, Automation

## Abstract

**Background:**

Over fifty percent of stroke patients experience chronic arm hand performance problems, compromising independence in daily life activities and quality of life. Task-oriented training may improve arm hand performance after stroke, whereby augmented therapy may lead to a better treatment outcome. Technology-supported training holds opportunities for increasing training intensity. However, the effects of robot-supported task-oriented training with real life objects in stroke patients are not known to date. The aim of the present study was to investigate the effectiveness and added value of the Haptic Master robot combined with task-oriented arm hand training in chronic stroke patients.

**Methods:**

In a single-blind randomized controlled trial, 22 chronic stroke patients were randomly allocated to receive either task-oriented robot-assisted arm-hand training (experimental group) or task-oriented non-robotic arm-hand training (control group). For training, the T-TOAT (Technology-supported Task-Oriented Arm Training) method was applied. Training was provided during 8 weeks, 4 times/week, 2× 30 min/day.

**Results:**

A significant improvement after training on the Action Research Arm Test (ARAT) was demonstrated in the experimental group (p = 0.008). Results were maintained until 6 months after cessation of the training. On the perceived performance measure (Motor Activity Log (MAL)), both, the experimental and control group improved significantly after training (control group p = 0.008; experimental group p = 0.013). The improvements on MAL in both groups were maintained until 6 months after cessation of the training. With regard to quality of life, only in the control group a significant improvement after training was found (EuroQol-5D p = 0.015, SF-36 physical p = 0.01). However, the improvement on SF-36 in the control group was not maintained (p = 0.012). No between-group differences could be demonstrated on any of the outcome measures.

**Conclusion:**

Arm hand performance improved in chronic stroke patients, after eight weeks of task oriented training. The use of a Haptic Master robot in support of task-oriented arm training did not show additional value over the video-instructed task-oriented exercises in highly functional stroke patients.

**Clinical trial registration information:**

Current Controlled Trials ISRCTN82787126

## Background

Stroke is a leading cause of morbidity worldwide and the first cause of motor impairment [1,2]. Chronic arm hand performance problems are present in over 50% of the stroke patients [3], limiting the use of their arm and hand in everyday life activities [4,5], but also limiting engagement in social life, and quality of life in general [6].

Rehabilitation may have positive effects on the improvement of arm hand performance [7], in acute as well as in chronic stages after stroke [8]. It is also known that a higher therapy intensity and duration leads to a better therapy outcome after stroke [9,10]. However, because of the increasing incidence of stroke [11], the availability of augmented therapy will be compromised. Technology-supported training is emerging as a solution to support therapists in their efforts and to relieve pressures on the health system.

Initial results of meta-analyses on clinical trial results with robotics only showed the effectiveness of robotics for the improvement of upper extremity function (muscle strength, coordination, joint range of motion), and not for the improvement on the ICF activity level (arm hand skilled performance) [7,12-14]. One of the reasons for the absence of activity-related training effects, was the absence of activity related training input [12-14]. Recently, more trial results have been published with positive effects of robot supported arm training on the ICF activity level outcome measures, e.g. a randomized clinical trial using T-WREX [15], and MIT Manus [16] in stroke, and the most recent systematic review of Mehrholz J. et al. [17] also found positive effects of the use of robotics and electromechanical devices on the upper extremity activity performance after stroke. But also in other neurological pathologies, such as MS, positive effects on the activity level after the use of robotics have been registered [18,19]. The study of Carpinella et al. [19] uses task oriented training with object manipulation, finding additional effects over reaching without object manipulation in MS patients.

While French et al. [20] did not find evidence for the effectiveness of task-oriented training of the upper extremity in stroke, results of clinical trials with technology supported task training look more promising. Several authors [21-24] have combined the task-oriented training method using real life objects with sensor-based technologies for the rehabilitation of arm hand performance after stroke. Results from pilot studies show an improvement of arm hand function [21], arm hand activity [21,25], and health related quality of life [21] after stroke. But also treatment credibility and patient motivation are quite high for such training approaches [22]. Johnson et al. [26] were the first to combine the task-oriented training approach using real life objects with the Haptic Master robot in stroke patients, enabling the handling of real life objects during robot training. Carpinella et al. [19] used the Braccio di Ferro robot in combination with a functional orthosis, allowing for the execution of functional tasks with real life objects in MS patients. However, no results on clinical effectiveness of task-oriented robot supported training in stroke patients are available to date. Also, while robot-supported training has been extensively investigated and proven effective in chronic stroke patients with lower functional levels [16,27], to the authors knowledge no evidence is available on the use of robot supported training in highly functional chronic stroke patients.

The aim of the present study was to investigate the effectiveness and added value of the Haptic Master robot adjunct to task-oriented arm hand training in chronic stroke patients on the ICF body function level, the ICF activity level, and on the quality of life.

## Methods

### Participants and study protocol

Twenty-two chronic stroke patients were recruited from Adelante Rehabilitation Centre (Hoensbroek, NL) to participate in a single-blind RCT. The sample size was determined after a power calculation based on results of a sensor-based intervention that used the same training method as the one in this study [21].

Inclusion criteria were 1) first ever stroke, 2) age between 18–85 years 3) clinically diagnosed with a central paresis of the arm/hand (strength: Medical Research Council grade 2–4 at entry into study), 4) post-stroke time ≥ 12 months, 5) fair to good cognitive level (Mini Mental State Examination (MMSE) score ≥ 26 [28]), 6) able to read and understand the Dutch language, 7) unable to fully perform at least two of the following skills: drinking from a cup, eating with knife and fork, taking money from a purse and using a tray, 8) motivated to train at least two of the abovementioned skills. At the start of the last 6 months of the inclusion period, inclusion criterion 4 was adjusted to post-stroke time ≥ 8 months, to facilitate patient inclusion. Exclusion criteria were: 1) severe neglect (Bell Test [29], Letter Cancellation Test: minimum omission score of 15% [30]), 2) hemianopsia, 3) severe spasticity (Modified Ashworth Scale total arm > 3), 4) severe additional neurological, orthopaedic or rheumatoid impairments prior to stroke which could interfere with task performance, 5) Broca aphasia, Wernicke aphasia, global aphasia (determined by the Akense Afasie Test [31], 6) apraxia (apraxiatest of Van Heugten [32]) and 7) attending another study or therapy to improve arm-hand function.

The participating rehabilitation physicians identified potential participants based on their medical files. Letters with information about the study and an invitation to participate were sent to potential participants. After receiving informed consent, the rehabilitation physician screened the patients willing to participate as to the inclusion and exclusion criteria. After inclusion, participants were randomly allocated to either the experimental group (robot-assisted training group) or the control group, using blocked randomization (block size = 2). The randomization procedure was performed by an independent researcher using 2 opaque envelopes with in each envelope a training condition code. Persons involved in data collection were blinded for group allocation. During the study period, participants were asked not to participate in other studies involving arm-hand performance and not to change the therapy they received next to this intervention. Participants were reimbursed for transportation costs to and from the rehabilitation centre. All procedures were approved by the Medical Ethics Committee of Adelante. All participants signed an informed consent prior to participating in the study. A written informed consent was also obtained for publication of the patient photos for scientific purposes. This trial was registered as ISRCTN82787126.

### Task-oriented training method and robotic system

**Figure 1:**
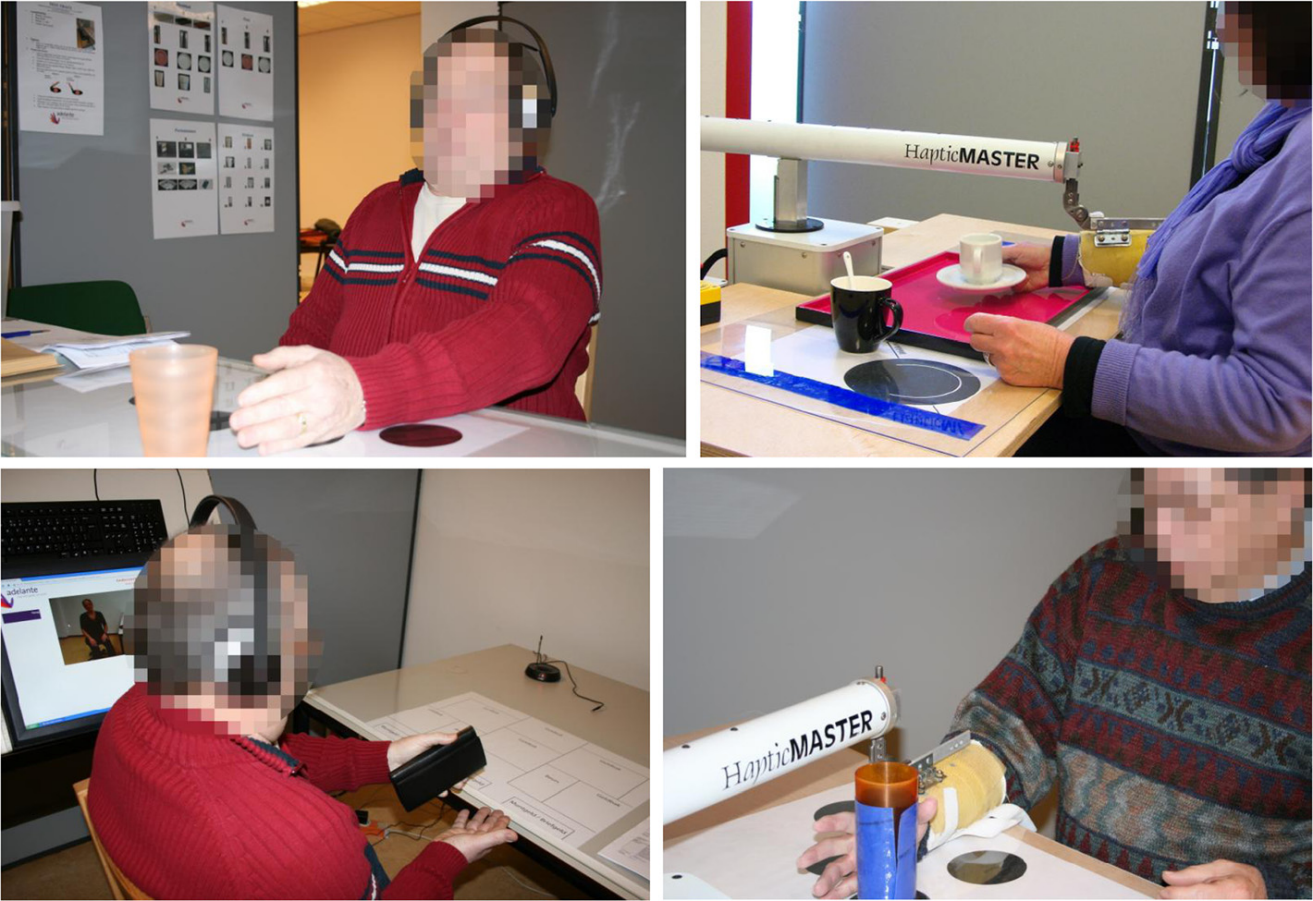
Picture of patients training in the control group (left) and in the experimental group (right).

The robotic system Haptic Master (MOOG, Nieuw-Vennep, NL) was used in the present study for the arm-hand training in the experimental group (Figure 1). The Haptic Master is a commercially available end-effector based robot that permits training of real-life functional tasks involving reach, grasp, as well as object transportation in a three dimensional space. The environment is set-up to support seated as well as standing up task performance. The Haptic-Master and the currently used gimbal allow six degrees of freedom (DoF). Three actuated (i.e. activated by the system) DoFs are for positioning and three non-actuated DoF’s are for orientation in the gimbal. This configuration permits the person to freely orient their hand as needed to manipulate an object.

For training, the T-TOAT (Technology-supported Task-Oriented Arm Training) method [33] was applied. The training method comprises of breaking down skills into functional components that maintain a strong relationship with the original skill itself. For each of these components exercises are offered at gradually increasing levels of difficulty, based on progress criteria from the fields of exercise physiology [34], and motor learning [35]. With regard to principles from exercise physiology, patients were instructed to not only focus on training coordination with low weight objects, but also include series where a number of repetitions was performed with heavier objects, in order to train on endurance (e.g. 50% of maximal weight, 3×15 repetitions) and strength (e.g. submaximal weights, 8–10 repetitions) [36]. With regard to motor learning, treatment variability was encouraged as patients were asked to use as many different objects as possible (e.g. different shapes and sizes of cutlery, different kinds of cups and glasses, several shapes and sizes of purses, different coins, etc.). Also patients were explicitly asked to mix different exercises and different tasks as random practise is known to support motor learning and retention of training effects through high contextual interference [37,38]. Participants are encouraged to first train on components of a skill (e.g. reach out to cup, grasp cup, lift cup, bring cup to mouth, empty cup in mouth, place cup on table), after which the complete action (e.g. drinking from a cup), sequencing all components, is trained. The “Haptic-TOAT” software tool [33] was used to enable the use of the T-TOAT method in combination with the Haptic Master robot. Individual movement trajectories can be recorded for each patient, in order to train on the for each patient optimal path. The 3D positions (x,y,z coordinates) are logged with a sample rate of 100 Hz, and can be saved and replayed. The number of repetitions can be adjusted per patient. Several modes can be used: the passive mode (for patients with very little strength or for learning the movement trajectory), or the active mode. In the passive mode, the trajectory is covered by the Haptic Master, taking the patient’s arm along the recorded path. In the active mode, the patient performs the arm movement along the recorded trajectory, whereby a deviation from the trajectory is corrected by bouncing into a wall, the diameter of the tunnel around the trajectory can be set by the therapist (a larger diameter is more difficult). Also correction of trajectory deviation is supported by a spring, with customizable strength, that pulls the robot end effector (and thus the patient arm) back to the optimal movement trajectory (stronger spring = easier). In addition to the spring force, damping (allowing for strength training) and addition of a force in the vertical direction (either supporting the arm against gravity or addition of extra load, both possible at the wrist and distal part of the forearm via the orthotic arm-HM interface) may be set at each point of the trajectory with the same graphical user interface. The T-TOAT method and its use with the Haptic Master are extensively described in a paper of Timmermans et al. [33].

### Arm-hand training program

Training was provided during 8 weeks, 4 times/week, twice a day for 30 minutes (separated by 0.5 hour to 1 hour of rest). At the start of the training program participants in both groups chose a minimum of 2 out of 4 skills to train: ‘drinking from a cup’, ‘eating with knife and fork’, ‘taking money from a purse’ or ‘using a tray’. The reason for letting patients choose the tasks they preferred most, was that we wanted to provide in both groups, to some extent, goal oriented training where patients would train towards personally set goals. This to enhance patient compliance, patient motivation and self-efficacy [39-41]. The tasks for which exercises were provided are chosen out of a list of training preferences for stroke patients [42]. Before training, participants were educated about the principles of task-oriented arm-hand training and the importance of frequent training to enhance therapy success. Video-instructions were used to explain the exercises. The video-instructions were organised per task, and within the task per skill component. Patients could easily select by themselves the appropriate training content. For each skill component at least 5 exercises were given in increasing difficulty level. The training program was similar for the experimental group and the control group, the only difference being the use of technology in the experimental group. Both groups received the same video-instructions, together with a tool box filled with training objects and posters how to use different objects with increasing difficulty levels (e.g. round glass with anti-slip material → normal glass→ wine glass). The exercises were done by (dis)placing objects on pre-printed templates, which were the same in both groups. The experimental group, by combining the T-TOAT training with the use of a Haptic Master robot received trajectory guidance through haptic feedback; while the control group had to master all upper extremity movements without support. Training in both, the experimental and control group, was supervised by a physiotherapist, occupational therapist or movement scientist. The therapist was present during the training (for both groups) to answer questions of the patients when necessary, to assist with advice on the use of objects, or to provide assistance in adaptations of the Haptic TOAT software. Assistance or resistance during training was adjusted between training sessions, by changing the strength of the spring force or damping in the Haptic Master software, in order to always maximally challenge the patient according to his/her potential. The patients were offered exercises that they could just about manage successfully without an increase of their compensatory strategies during movement performance. This approach is supportive of motor learning in stroke patients [33]. The therapists used the assistance and damping facilities in the software according to their clinical experience. No recordings were made of how therapists actually used these training facilities. Both groups received equal attention of the therapist. Compliance was determined as the percentage of training sessions patients attended relative to the maximal total of 64 training sessions. Patients continued their usual care outside of this intervention. The usual care therapy did not consist of arm-hand activities at the ICF activity level.

### Outcome measures

The demographical data obtained from the medical files were, age, gender, date and type of stroke, side of hemiparesis, and hand dominance. Outcome measurements were taken upon entry into the study (T0, baseline), after 4 weeks training (T1, only for primary outcome measures), at the end of the 8 weeks training program (T2), and 6 months after finishing the training program (T3). A diary was kept during training sessions to determine therapy compliance and to record which exercises were practised as well as the number of repetitions.

Primary outcome measures consisted of assessment at the ICF body function level (Fugl Meyer Motor Assessment (FMMA) [43]), and at the ICF activity level (Action Research Arm Test (ARAT) [44], Motor Activity Log (MAL) [45]). The upper extremity section of the FMMA was used. FMMA has been found a reliable and valid test for the assessment of arm hand function in stroke patients [43]. The maximum score on the FMMA, upper extremity section is 66. The ARAT has been proven to be a reliable, valid and sensitive instrument for upper limb activity measurement [44-46]. The maximum score on ARAT is 57. The MAL is a semi-structured interview to assess frequency of use (AU) (“How often did you actually use your affected arm for this activity ?”, 6 point likert scale: 0 = not used/5 = same as pre-stroke) and quality of use (QU) (“How useful was the contribution of your affected arm during this activity?”, 6 point likert scale: 0 = no contribution/5 = same as pre-stroke) of the affected limb for skill performance. The MAL has been shown to be a reliable and valid tool for measurement of arm hand activity in stroke patients [47].

Secondary outcome measurement, at the ICF participation level, consisted of quality of life assessment (EuroQol-5D and SF-36). The EuroQol-5D (EQ-5D) is a broad generic assessment tool for quality of life [48]. It includes a Visual Analogue Scale (VAS) (0–100) to indicate the perceived health-state, as well as scoring of 5 sub-items (0–2 score) (mobility, self-care, usual activities, pain, and anxiety). The EuroQol-5D has good psychometric properties [49]. The SF-36 Health Survey (SF-36) [50] is a generic survey with a good reliability and validity. SF-36 is composed of 36 questions and standardized response choices, and yields physical and mental health summary measures. All raw scale scores were linearly converted to a 0 to 100 scale, with higher scores indicating higher levels of functioning or well-being [50]. SF-36 has a greater sensitivity than EQ-5D, however EQ-5D shows higher completion rates [51].

Persons who performed the assessments, were not involved in training and data analysis. Intention to treat analysis was applied: data were analysed according to the original random assignments, regardless of whether the patients actually received treatment.

### Data processing and analysis

If between different test occasions only one of the test results was missing for a person, the missing value was substituted according to the last observation carried forward principle. No baseline data were missing.

As data did not follow a normal distribution pattern, non-parametric statistics were used.

Data were analyzed using SPSS software (SPSS Inc., Chicago, IL). For testing of differences between groups with regard to nominal data (side of hemiparesis, hand dominance), a Chi Square Test was performed. Other differences between the experimental and control group were tested with the Wilcoxon-Mann–Whitney *U* test. A Friedman analysis was performed to assess if significant progress was made over time (T0,(T1),T2,T3) within either one of the treatment groups. Alpha was set at 0.05. The progress made between two specific test occasions within a group was tested with a Wilcoxon signed ranks test, using a Bonferroni approach. The Bonferroni corrected alpha value equals 0.0125 (for data comparison T0-T2, T2-T3, T0-T1, T1-T2) [52].

## Results

**Figure 2:**
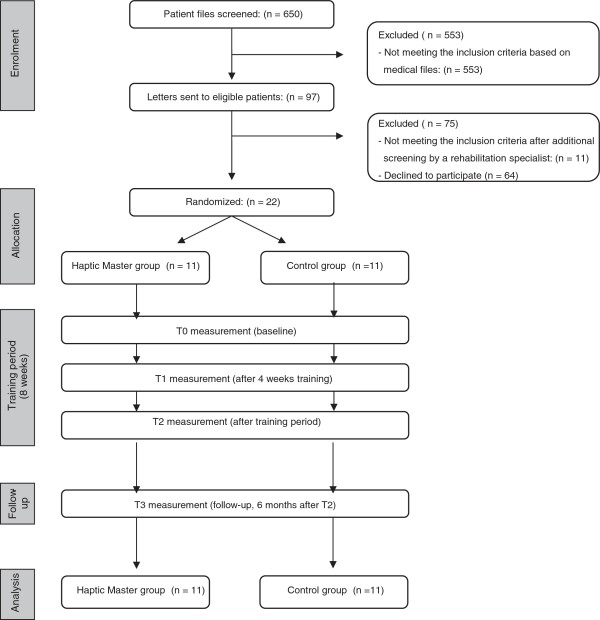
Flow chart representing the number of patients throughout the trial measures.

The inclusion of patients into the study started in May 2009 and lasted till May 2011. Figure 2 gives an overview of the trial profile, i.e. the number of participants throughout the study.

### Error analysis

As for the primary outcome measures, only 1 T3 measurement was missing from the FMMA data. As for the secondary outcome measures, only 1 T3 measurement was missing for the SF-36 test. Both were from the same patient who was not willing to stay as long as we needed to complete the test because of conflicting agenda items. Intention to treat analysis was applied. The missing T3 data were substituted by the T2 data from the same person.

### Patient characteristics

Participant characteristics are presented in Table 1. Five patients were included with a post stroke time shorter than the initially required 12 months (i.e. experimental group: 1× 10 months and 1× 8 months, control-group: 2× 11 months, 1× 10 months post-stroke). No significant differences were found for age and post-stroke time between the experimental group and the control group at baseline (p = 0.133, and p = 0.606 respectively). Also no significant differences were found for the side of hemiparesis (p = 0.08) and for dominant arm impairment (p = 1.0). For patient characteristics at baseline, only for hand dominance a significant difference was found between groups (p = 0.03). At baseline, arm-hand function did not differ significantly between patients in the experimental and the control group, as determined by the FMMA (p = 0.401), ARAT (p = 0.365) and MAL (p = 0.797). No patients dropped-out of the study. Compliance of the patients with attending the training scheme was 96% in the experimental group and 96.2% in the control group. One patient (in the experimental group) fainted briefly once. However, after visiting the medical specialist, the cause turned out to be a change of medication. No relationship with the intervention was found. No adverse effects of the study were found.

**Table 1:** Patient characteristics at baseline

	**Control group n = 11**	**Experimental group n = 1**	**Between group difference**
Mean age in years (SD)	56.8 (6.4)	61.8 (6.8)	p = 0.1
Gender			
Male	8	8	
Female	3	3	
Mean time post-stroke in years (SD)	3.7 (3.0)	2.8 (2.9)	p = 0.6
Side of hemiparesis			
Left	8	7	p = 0.08
Right	3	4	
Arm hand dominance			
Left	1	5	p = 0.03
Right	10	6	
Dominant arm hand impaired	4	7	p = 1.0
Non-dominant arm hand impaired	7	4	
MEdian [IQR] FMMA	53 [47, 57]	50 [39, 58]	p = 0.4
Median [IQR] ARAT	39 [28, 46]	31 [24, 40]	p = 0.3
Median [IQR] MAL	4.1 [2.4, 6.9]	3.7 [2.7, 4.9]	p = 0.7

*Abbreviations*: *SD* Standard deviation, *IQR* Interquartile range, *FMMA* Fugl Meyer Motor Assessment, *ARAT* Action Research Arm Test, *MAL* Motor Activity Log.

### Primary outcome measures

An overview of the test results can be found in Table 2. An overview of the individual improvement over time relative to baseline values (IIT) between the start of the training and the cessation of the training for the different tests can be found in Table 3.

**Table 2:** Results on primary outcome measures

	**Control group**					**Experimental group**				
	**T0 MED [IQR]**	**T1 MED [IQR]**	**T2 MED [IQR]**	**T3 MED [IQR]**	**P values**	**T0 MED [IQR]**	**T1 MED [IQR]**	**T2 MED [IQR]**	**T3 MED [IQR]**	**P values**
FMMA	53 [47, 57]	52 [44, 58]	54 [51, 59]	53 [50.7, 59.5]	NS	50 [39, 58]	54 [45, 59]	55 [46, 56]	52 [43, 59]	NS
ARAT	39 [28, 46]	43 [28, 49]	43 [32, 51]	50 [27, 54]	NS	31 [24, 40]	38 [24, 43]	34 [25, 41]	37 [25, 49]	** ^††^T0-T2
MAL										
AU	2.2 [1.5, 3.8]	2.8 [1.5, 4.6]	3.7 [2.5, 4.2]	3.8 [1.7, 4.3]	** ^††T0-T1^	2.2 [1.5, 2.8]	1.9 [1.4, 3.6]	2.2 [1.6, 4.2]	2.8 [2.2, 3.8]	ns
QU	1.8 [0.9, 3]	2.7 [1, 3.5]	3 [2.2, 3.5]	2.3 [1.3, 4]	** ^†† T0-T2 ††T0-T1^	1.5 [1.1, 2.1]	1.7 [0.9, 3.1]	1.6 [1.3, 3.4]	2 [1.6, 3.5]	** ^††T0-T2^
TOT	4.1 [2.4, 6.9]	5.3 [2.5, 8.3]	6.1 [4.8, 7.8]	6.1 [3, 8.5]	** ^††T0-T2 ††T0-T1^	3.7 [2.7, 4.9]	3.6 [3.6, 2.2, 7]	3.7 [3.6, 7.9]	5 [4, 7.4]	**
EQ-5D [VAS]	70 [64, 75]	-	78 [68, 90]	75 [60, 80]	NS	65 [63, 85]	-	80 [70, 80]	74 [70, 80]	NS
SF-36										
Physical health	59 [519, 65.4]	-	71 [59.4, 92]	64 [52.8, 84.3]	** ^††T0-T2 †† T2-T3^	58.4 [38.9, 64.9]	-	58.4 [55, 70]	64 [58.4, 77]	NS
Mental health	84.5 [74.8, 95.25]	-	87.6 [72.6, 98.7]	86.6 [75.2, 100]	NS	86.5 [81.6, 90.5]	-	86.7 [81.7, 91.3]	82.7 [76.3, 91.2]	NS

*Abbreviations*: *MED* Median, *IQR* Interquartil range, *FMMA* Fugl Meyer Motor Assessment, *ARAT* Action Research Arm Test, *MAL* Motor Activity Log, *AU* Amount of use, *QU* Quality of use, *TOT* total, *EQ-5D* EuroQol-5D, *VAS* Analogue Scale, *IIT* Individual improvement overtime between start and cessation of the training, relative to baseline values. ***Friedman p < 0.001, **Friedman p < 0.01, *Friedman p < 0.05, ††Wilcoxon p < 0.0125, NS = non significant.

**Table 3:** Individual improvement over time for the primary and secondary outcome measures

	**Control group IIT T0-T2 MED [IQR]**	**Experimental group IIT T0-T2 MED [IQR]**	**P-value**
**FMMA**	3.5 [0, 8]	1.6 [−5.1, 19.1]	0.51
**ARAT**	16.1 [0, 35.8]	9.0 [3.8, 18.7]	0.79
**MAL**			
AU	33.1 [9.6, 96]	9 [0.4, 51]	0.33
QU	46.5 [23, 78.2]	41.3 [14.28, 58.9]	0.4
TOT	44.9 [15.6,108.2]	24.3 [9.1, 55.2]	0.36
**EQ-5D**	11.4 [0, 25]	7.8 [−4.9, 24]	0.5
**SF-36**			
Physical health	22.7 [2.1, 26.7]	13.4 [−5, 32.4]	1.0
Mental health	0 [0, 8.4]	0 [−1.9, 8.3]	0.6

*Abbreviations*: *MED* Median, *IQR* Interquartile Range, *FMMA* Fugl Meyer Motor Assessment, *ARAT* Action Research Arm Test, *MAL* Motor Activity Log, *AU* Amount of use, *QU* Quality of Use, *TOT* total, *EQ-5D* EuroQol-5D, *IIT* Individual improvement over time between start and cessation of the training, relative to baseline values.

### Between group results

No between group differences were found with regard to arm hand function (FMMA), arm hand capacity (ARAT), and self-perceived arm-hand performance (MAL).

### Within-group results

No within-group differences were detected between different test occasions for FMMA, neither in the control group, nor in the experimental group.

With regard to the ARAT, a significant improvement over time occurred in the experimental group (p = 0.004). The progress was significant between the start of the training and the end of the training (p = 0.008). There was no difference found in ARAT results between cessation of the training and the 6-month follow-up measurement.

With regard to the MAL (total test result), a significant improvement over time was found in the control group (p = 0.002), and in the experimental group (p = 0.005). The progress was significant between the start of the training and the end of the training in the control group (p = 0.008), and a trend towards significance was observed in the experimental group (p = 0.013). Also the progress on the MAL was significant between the start of the training and the intermediate measurement for the control group (p = 0.004). No difference was found in either group between the cessation of the training and the 6-month follow-up measurement.

When looking at the MAL test results for AU and QU separately, it was found that for AU a significant difference was found in the control group over time (p = 0.008), and specifically between T0 and T1 (p = 0.008). With regard to the MAL test results for QU, a significant improvement over time was found for the control group (p = 0.001) and for the experimental group (p = 0.006). Also significant differences were found for QU in both groups between start and cessation of the training (control group p = 0.006, experimental group p = 0.01). For the control group, also significant differences in QU were found between T0 and T1 (p = 0.004).

No significant differences were found between cessation of training and the follow up measurement for any of the primary outcome measures.

### Secondary outcome measurements

An overview of the test results can be found in Table 2. An overview of the individual improvement over time (IIT) between the start of the training and the cessation of the training for both quality of life measurements can be found in Table 3.

### Between group results

A significant difference was found between the control and experimental group with regard to the delta physical health scores of the SF-36 T2-T3 (p = 0.002). In the control group a deterioration in perceived physical health scores was observed, while in the experimental group physical health scores improved after cessation of the training. No other between group differences were found between the control and the experimental group with regard to quality of life, neither for EQ-5D results, nor for SF-36 results.

### Within-group results

In the control group, a trend for significant improvement in the EQ-5D health status (VAS) and a significant improvement in the SF-36 physical health component was found between start and cessation of the training (EQ-5D: p = 0.015; SF-36 physical health, p = 0.01).

For the control group, a significant decrease was shown in physical health scores after cessation of the training until 6 months follow up (p = 0.012).

No within-group differences were found between test occasions for the different sub-items of the EQ-5D, nor for the items of SF-36 relating to mental health.

## Discussion

The aim of the present study was to investigate the effectiveness and added value of the use of the Haptic Master robot adjunct to task-oriented arm hand training in chronic stroke patients on the ICF (International Classification of functioning, disability and Health) function level, the ICF activity level, and on the quality of life.

No differential effects could be demonstrated between the control and experimental group on any of the primary outcome measures. The highly functional chronic stroke patients in this study did not seem to benefit from the addition of a Haptic Master robot to execute task-oriented training with real life objects over training with the same video instructed exercises and real life objects alone. Hesse et al. have argued that stroke patients with lower functional levels may benefit more from robot-supported training where actuator assistance to movement may overcome problems, such as muscle weakness [53]. In these patients, the robot assistance is of great importance for obtaining the critical number of repetitions that contribute to motor learning and to improvement of arm hand performance, while higher functional patients may obtain the critical number of repetitions for improvement without robot assistance. On the other hand, an additional effect could be expected from the haptic feedback in the robot supported training, as feedback is known to be an important component for motor learning [54]. Especially haptic feedback can bring an additional and more immersive sensation than purely visual environments, thereby increasing the person’s attention and motivation during repeated performance [55].

Although no differential effects were found, it should be emphasized that in the current trial, both groups improved with regard to activity based arm hand performance measures, indicating that the task-oriented training method with real life objects that was used in both groups resulted in the improvement of activity based motor performance, rather than the robot support. On Fugl Meyer no statistically significant improvements were found in either group, and also individual improvement over time was smaller than 4% (Table 3). This result can be explained by training specific effects of the activity based training approach in both groups. Almost all results on individual improvement over time (Table 3) of ARAT and MAL were above 10%. The results of this study corroborate the findings of Lo et al. [16] and of Kahn et al. [56] who found that in patients with long-term upper limb deficits after stroke, robot-assisted therapy had no additional effects over non-technology supported control interventions. But, as in our study, Lo et al. [16] and Kahn et al. [56] did find evidence for gains in upper extremity motor function after arm hand training in chronic stroke patients.

In our study only the experimental group improved statistically significantly on the ARAT, which is a capacity test [57] on the ICF activity level. The ARAT is mostly focusing on grasping and displacing several objects in a confined workspace, whereby successful grasp and displace movements, as well as quality of movement performance with regard to the compensatory shoulder girdle and arm movements during grasp, are evaluated. Patients in the experimental group received haptic guidance as to following the correct movement trajectory, thereby allowing them to focus on correct movement trajectories during a grasp and displacement task. The statistically significant improvement on the ARAT in the experimental group may be explained by Haptic Master guidance, leading to a) better ARAT results as patients in the Haptic Master group were able to focus on correct trajectory performance guided by extra proprioceptive feedback from the Haptic Master; and b) a reduced within group variability in treatment results due to more standardized exercise performance, which is corroborated by the lower IIT values on ARAT for the experimental group compared with the control group (Table 3). The improvements on the ARAT in the experimental condition alone, may therefore be attributable to training specific effects of the Haptic Master.

As for perceived arm hand performance (measured by the MAL), both groups showed significant improvements after training, that were maintained until 6 months after the training had stopped. Whereas the ARAT measures the highest level of performance in a test situation, the MAL measures the functioning in daily life situations as perceived by the patient [58]. The perceived individual improvement over time with regard to daily life performance is lower for the experimental group (see Table 3). This may be attributed to the fact that the activity training in the control group was more similar to performance of everyday life activities than the activity training of the experimental group, leading to better performance in everyday life and/or more confidence for the performance of everyday life activities. In the control group a significant improvement in health related quality of life (SF-36) was found. This could be related to the increase of perceived performance (IIT MAL was higher in the control group, see Table 3) in the everyday life situation. However, these gains could not be maintained at 6 months after cessation of the training.

### Methodological considerations

Interactive systems that guide and support motor actions depending on the needs of the patient, offer opportunities for motor learning through the prolonged active involvement of the patient in a high number of exercise repetitions, and through reducing effort and fatigue [55,59]. It is indeed a strength of robotic systems to allow for a high number of exercise repetitions, which is known to contribute to more improvement of arm hand performance after stroke [9]. Because it did take some time (approximately 3 minutes for new recording, 1 minute when old recording was used) to start up and adjust the settings for each exercise in the Haptic TOAT software environment, and because of the confined total exercise duration (2 × 30 min/day), the current intervention may not have made optimal use of the opportunity to reach a high number of exercise repetitions. Given these potential influences, software optimization and unlimited training times could have resulted in higher training effects in the same period of time.

The improvement on test results of the Motor Activity Log was quite high in both groups. The Motor Activity Log is a self-evaluation scale. Because of the effort participants in this study have invested into the training, high expectations of the participants for the treatment outcome may have influenced the results of these self-appraisal scales [60].

It could be argued that our study may have been underpowered, which may have contributed to the lack of significance between groups. Only 21% of the eligible patients were willing to participate in this study. The most important reason for not participating was the length and the intensity of the training period. This may have lead to a selection bias towards recruitment of persons with a high level of motivation to train, which may have influenced the results in both, the control and the experimental group.

The patients included in this study had a relatively high functional level (see Table 1). It is not known whether the results of this study can be generalized to patients with more severe impairments.

### Considerations for future research

It would be very interesting to repeat this trial for low functional stroke patients, in order to assess and compare the benefit of Haptic Master supported training across a wider spectrum of stroke patients. It can be expected that patients with a lower functional level may benefit more from robot-assistance to movements that they cannot perform actively. They may also benefit more from the minimization of execution errors through haptic guidance that has shown to be beneficial for motor learning [61].

## Conclusion

Arm-hand performance improved in highly functional chronic stroke patients, after eight weeks of task oriented training, in both the robot- and in the non-technology supported intervention. However, the use of a Haptic Master robot in support of task-oriented arm training did not seem to have an additional value over the video-instructed task-oriented exercises.

## Competing interests

The contributing authors guarantee that this manuscript has not been submitted, nor published elsewhere. Each of the authors declares that he/she does not have any financial or non-financial competing interests.

## Authors’ contributions

All authors have proofread the manuscript and agree with the final manuscript version. AT, HS, WB, and RG participated in the conception and design of the study. MM, RL, AT participated in data collection. RS, HS, AT, RL have been involved in data analysis and interpretation and also in drafting and writing of the manuscript.
